# Novel Insights into the Molecular Mechanisms Governing Mdm2 Ubiquitination and Destruction

**DOI:** 10.18632/oncotarget.202

**Published:** 2010-10-20

**Authors:** Hiroyuki Inuzuka, Hidefumi Fukushima, Shavali Shaik, Wenyi Wei

**Affiliations:** Department of Pathology, Beth Israel Deaconess Medical Center, Harvard Medical School, Boston, MA 02215

**Keywords:** Ubiquitination, SCF, β-TRCP, Casein Kinase I, Mdm2, p53, DNA Damage, Phosphorylation, Cell Cycle

## Abstract

The Mdm2/p53 pathway is compromised in more than 50% of all human cancers, therefore it is an intensive area of research to understand the upstream regulatory pathways governing Mdm2/p53 activity. Mdm2 is frequently overexpressed in human cancers while the molecular mechanisms underlying the timely destruction of Mdm2 remain unclear. We recently reported that Casein Kinase I phosphorylates Mdm2 at multiple sites to trigger Mdm2 interaction with, and subsequent ubiquitination and destruction by the SCF^β-TRCP^ E3 ubiquitin ligase. We also demonstrated that the E3 ligase activity-deficient Mdm2 was still unstable in the G1 phase and could be efficiently degraded by SCF^β-TRCP^. Thus our finding expands the current knowledge on how Mdm2 is tightly regulated by both self- and SCF^β-TRCP^-dependent ubiquitination to control p53 activity in response to stress. It further indicates that loss of β-TRCP or Casein Kinase I function contributes to elevated Mdm2 expression that is frequently found in various types of tumors.

## INTRODUCTION

Recent scientific advances clearly demonstrated that tumors arise in a progressive fashion through overexpression of certain set of oncogenes, concomitantly with loss of function mutations in key tumor suppressor proteins. Among the known tumor suppressor proteins, the p53 tumor suppressor attracts intensive research interest since its activity is lost or compromised in over 50% of all human tumors [1]. p53 exerts its tumor suppressor function mainly through its role as a transcription factor to activate a wide spectra of downstream target genes. The activation of these downstream targets in turn are responsible for p53-dependent cell cycle arrest or apoptosis upon a variety of cellular stresses [2,3]. Among the ever-growing list of p53 targets, key players include p21, which initiates both G1 and G2 cell cycle arrest by inhibition of cyclin-dependent kinases [4,5]; Gadd45 and 14-3-3, which participate in G2 cell cycle arrest [6,7]; and Bax, which is responsible for the pro-apoptotic activity of p53 [8]. Different types of DNA damage signals and variations in the extent of DNA-damage triggers activation of a distinct set of p53 downstream targets, thus initiating different types of stress response [9]. Because of its crucial role in response to DNA damage, p53 is considered the guardian of the genome whose activity is critical for maintaining the integrity of the genome [10,11].

### Mdm2 is the major negative regulator of the p53 pathway

**Figure 1:**
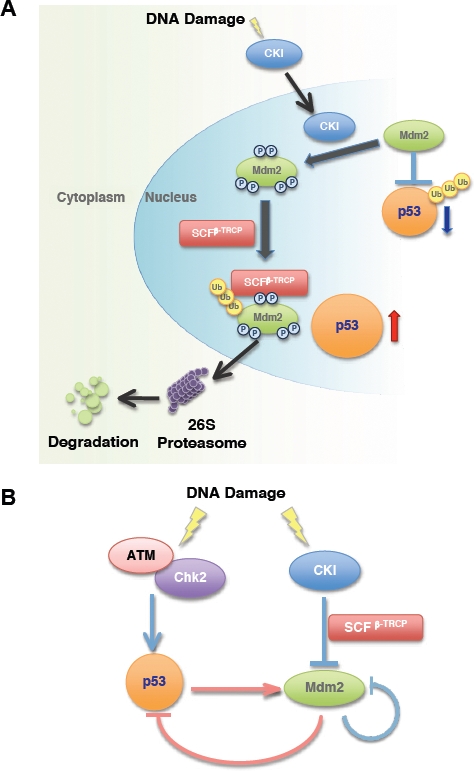
Multi-site phosphorylation of Mdm2 by Casein Kinase I triggers Mdm2 ubiquitination and destruction by SCF^β-TRCP^ in response to DNA damage **A.** In unstressed cells, p53 expression is maintained at basal level due to its interaction with Mdm2, which serves to promote p53 ubiquitination and subsequent destruction. In response to DNA damage, Casein Kinase I (CKI) translocates into the nucleus and phosphorylates Mdm2 at multiple sites. The phosphorylated Mdm2 species are recognized and ubiquitinated by SCF^β-TRCP^, and subsequently degraded by the 26S proteasome-dependent pathway. **B.** p53 phosphorylation by ATM/Chk2 and Mdm2 phosphorylation by CKI positively regulate the p53 pathway. Phosphorylation of p53 by ATM/Chk2 triggers the dissociation of p53 from Mdm2 and stabilizes p53. However, increased p53 activity induces Mdm2 transcription, which serves as a negative feedback loop to induce p53 downregulation. On the other hand, in response to the genotoxic stress, the Mdm2 oncoprotein is quickly degraded by the CKI/SCF^β-TRCP^ signaling pathway.

p53 activity is strictly regulated in cells to prevent inappropriate activation [12], which could result in either premature senescence or elevated apoptosis [13,14]. Among all the identified negative regulators, Mdm2 plays a critical role in promoting p53 ubiquitination and subsequent destruction (Figure 1A) [15,16]. The physiological significance of this layer of regulation is demonstrated by the fact that Mdm2 knockout mice are embryonic lethal due to elevated p53 expression and this lethality can be rescued by further inactivation of p53 [17].

Earlier studies showed that the inhibitory function of Mdm2 towards p53 relies mainly on its interaction with the p53 protein, a process that is subject to many layers of regulation (Figure 1B). In response to stresses such as DNA damage, activation of the ATM/ATR/CHK kinase pathway results in p53 phosphorylation at the Ser15 and Ser20 sites, which serve to disrupt the interaction between Mdm2 and p53. In this setting, phosphorylated p53 can escape Mdm2-mediated proteolysis and accumulate to sufficient levels to initiate the stress response checkpoints [18,19]. Previous work indicated that Mdm2, the major negative regulator of p53, was also quickly degraded in response to DNA damage signals [20]. Hence the destruction of Mdm2 is essential to allow p53 to become stabilized and fully activated. However, the molecular mechanisms accounting for the rapid destruction of the Mdm2 protein following the DNA-damage response still remains unclear.

### Self-ubiquitination is not required for Mdm2 destruction

Although it was previously proposed that Mdm2 undergoes self-ubiquitination when cells are treated with DNA damaging agents [20,21], a recent study utilizing transgenic mice elegantly illustrated that the E3 ligase activity of Mdm2 may not be required for the destruction of the Mdm2 protein [22]. We also obtained experimental evidence showing that the ring-finger mutant Mdm2, which is defective in its E3 ligase activity, is still unstable in the early G1 phase and is quickly degraded after DNA damage. These results strongly suggest that the destruction of the Mdm2 protein, which is central in regulating the p53 pathway, is tightly controlled by a foreign E3 ligase.

### The SCF^β-TRCP^ E3 ubiquitin ligase mediates Mdm2 destruction

We recently reported a novel molecular mechanism by which CKI-mediated phosphorylation of Mdm2 at multiple sites triggers β-TRCP-mediated Mdm2 destruction (Figure 1A and 1B) [23]. Furthermore, we demonstrated that this mechanism operates both in response to DNA damage stress and in normal cell cycle progression, thus tightly controlling the abundance of Mdm2. As a result, Mdm2 expression is low in the early G1 phase, and becomes elevated when cells enter the S phase [24]. When endogenous β-TRCP was depleted, Mdm2 abundance was mainly upregulated in the mid-late G1 phase. Correspondingly, the expression levels of p53 and its substrates p21 and Bax were reduced [23]. This indicates that β-TRCP-mediated Mdm2 destruction mainly operates in the mid-late G1 phase and that the fluctuation of Mdm2 might play an important role in cell cycle regulation through influencing the p53/p21 pathway.

It is well recognized that most F-box proteins including β-TRCP, only interact with their substrates once they are properly phosphorylated [25]. Therefore, the regulation of substrate destruction mainly occurs at the level of the modifying enzyme, and for the case of Mdm2, we found that the activity of CKI determines the timing for Mdm2 destruction. In support of this notion, blocking CKI activity with a widely-used pharmaceutical inhibitor [26] resulted in a marked increase in the steady-state level of Mdm2 [23]. Previous research demonstrated that CKI activity is subject to many layers of regulation. For example, Wnt signaling has been shown to positively regulate CKI [27] while PKA is a repressor of CKI activity [28]. Additionally, autophosphorylation at the C-terminus by CKI attenuates its own kinase activity [29]. Our studies indicate that CKI activity might be relatively higher in the mid-late G1 phase, although the precise molecular mechanism is still unclear.

### The CKI signaling pathway is also involved in DNA damage-induced Mdm2 destruction

In agreement with previous studies, we found that Mdm2 destruction was enhanced in response to DNA damaging treatment [20,23]. We also accumulated experimental evidence supporting the hypothesis that both CKI and β-TRCP are involved in this process. First, inactivation of β-TRCP by siRNA treatment partially blocked Mdm2 destruction after treatment with various DNA damaging agents. Secondly, inactivation of CKI by a widely-used pharmaceutical inhibitor blocked DNA-damage induced Mdm2 destruction. Furthermore, Mdm2 mutants whose putative CKI sites have been deleted displayed elevated levels of resistance to DNA damage-induced proteolysis. Although it was reported previously that CKI kinase activity is activated by DNA damaging signals [30], it is unclear how CKI is involved in controlling Mdm2 destruction after DNA damage. Our results argue that endogenous CKI activity was not significantly altered after DNA damaging treatment (data not shown). However, we found that DNA damage led to increased nuclear accumulation of CKIδ, which correlates with enhanced association between Mdm2 and CKIδ. These data indicate that DNA damage primarily modulates the accessibility of CKIδ cellular localization rather than its kinase activity to influence Mdm2 stability (Figure 1A) [23].

### CKI-mediated multi-site phosphorylation of Mdm2 promotes its destruction by SCF^β-TRCP^

**Figure 2:**
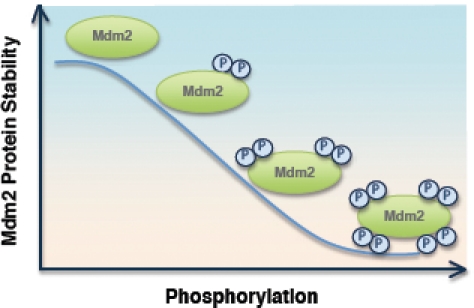
Inverse correlation between the extent of Mdm2 phosphorylation status and Mdm2 protein stability CKI-mediated multi-site phosphorylation of Mdm2 determines Mdm2 stability in cells.

Using both mass spectrometry analysis and *in vitro* kinase assays, we demonstrated that the major CKI phosphorylation sites are located in the Mdm2 acidic domain (p2), while other minor CKI sites are located in the N-terminal DSG site (p1) and the C-terminal SQ cluster (p3) region. In a search for major phosphorylation sites within those regions, we found that CKI phosphorylates Mdm2 at multiple sites, but we were not able to find any single point-mutation mutant that severely impaired CKI phosphorylation in comparison with the various deletion constructs. Interestingly, the loss of CKI phosphorylation in various Mdm2 mutants correlated very well with their reduced binding affinity, and subsequently their reduced destruction efficiency by β-TRCP1 [23]. This suggests that in contrast with other known β-TRCP substrates such as Emi-1 or Claspin, which contains one optimized canonical degron sequence, Mdm2 contains many suboptimized degron sequences that could be phosphorylated by CKI (Figure 2). It was found recently that many β-TRCP substrates including Cdc25A [31] and Gli3 [32-34] share the same feature by containing multiple degron sequences. A similar mechanism was first described for SCF^Fbw7^ to degrade its well-characterized substrate Sic1. Sic1 contained nine suboptimized degron sequences and phosphorylation of at least six of them are found to be required for initiating the destruction process [35]. It was further proposed that this multi-step phosphorylation might be a general mechanism to set a threshold for protein-protein interaction and thus create a strict switch-like transition [36]. Here, our studies suggested that the cumulative, multisite phosphorylation of Mdm2 by CKI might set up a “dimmer switch” for Mdm2 destruction in response to DNA damage (Figure 2) [23].

### Both Mdm2 self-ubiquitination and β-TRCP-mediated ubiquitination contributes to Mdm2 destruction

Mdm2 self-ubiquitination has been reported to play an important role in DNA-damage triggered Mdm2 destruction [20]. However, consistent with a recent report [22], we found that the ring-finger mutant (C464A) Mdm2 could still be efficiently degraded in response to various DNA damaging treatments. More importantly, ectopic expression of CKI and β-TRCP1 could efficiently destroy both the C464A mutant Mdm2 and the wild-type Mdm2, indicating the ring-finger activity of Mdm2 is not required for this process [23]. On the other hand, additional deletion of the p2 or p3 region greatly impaired β-TRCP-induced destruction of both wild-type Mdm2 and the C464A-Mdm2 mutant. These findings strongly imply that CKI-mediated phosphorylation, rather than the ring-finger domain of Mdm2, is critical for Mdm2 destruction after DNA damaging treatment.

On the other hand, we found that Mdm2 self-ubiquitination played a critical role in regulating the steady state level of Mdm2 in cell cycle progression. This is demonstrated by the observation that the C464A Mdm2 mutant was more stable than wild-type Mdm2 during the cell cycle progression, especially in the mid-late G1 phase [23]. Interestingly, we found that depleting endogenous β-TRCP led to further upregulation of the C464A-Mdm2 protein. On the other hand, the Mdm2 mutant defective in CKI phosphorylation was much more stable as well, and its expression was not responsive to depletion of endogenous β-TRCP. Taken together, these data indicate that constitutive Mdm2 destruction during the cell cycle progression is tightly regulated by both self-ubiquitination and the β-TRCP-mediated ubiquitination pathways [23]. However, as our data suggested, the β-TRCP pathway might play a more important function in triggering Mdm2 degradation after stresses such as DNA damage.

### Loss of function mutation in either CKI or β-TRCP might contribute to Mdm2 elevation in tumors

**Figure 3:**
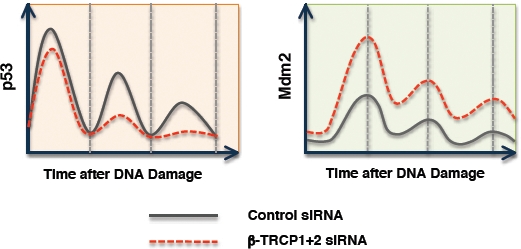
Depletion of endogenous β-TRCP results in elevated Mdm2 levels, which subsequently suppress the amplitude of the p53 pulse in response to DNA damage This further suggests that disruption of the CKI/SCF^β-TRCP^ signaling pathway, which governs Mdm2 ubiquitination and degradation, might lead to misregulation of the p53 activity that contributes to cancer development.

The Mdm2 protein has been characterized as an oncoprotein whose overexpression enhances cellular transformation. It is also widely accepted that Mdm2 is overexpressed in many types of tumors [37]. Although amplification has been found in many cases, the molecular mechanisms leading to elevated Mdm2 expression still remain unclear. Our studies suggested that dysfunction of the Mdm2 destruction pathway could potentially contribute to Mdm2 upregulation. This could be achieved by either inactivation of the responsible E3 ligase β-TRCP1 or by inactivation of the upstream modifying enzyme CKI. Alternatively, it could also be achieved by acquiring Mdm2 mutations that disrupt one or several of the multi-site phosphorylation events by CKI. Indeed, deletion of β-TRCP1 has been reported in many types of cancers [38,39]. Our data demonstrated that inactivation of β-TRCP1 resulted in significant upregulation of Mdm2, and subsequent downregulation of p53 activity. As a result, the cells are more resistant to DNA-damage induced apoptosis. Furthermore, our studies demonstrated that after depletion of β-TRCP, the oscillation of p53 expression in response to persistent DNA damage is affected (Figure 3) [23]. This result provided the molecular mechanism by which p53 activity can be dampered after depletion of β-TRCP. Altogether, this work suggests that a compromised Mdm2 destruction pathway might lead to subsequent Mdm2 stabilization, thus enhancing Mdm2 oncogenic activity by promoting p53 destruction and facilitating tumor progression. Therefore, our research provides insight into the underlying molecular mechanisms for the frequently observed Mdm2 overexpression in many types of tumors. This opens new avenues for the development of new anti-cancer drugs or regimens for treating cancer patients, especially those with elevated expression of the Mdm2 oncoprotein.
